# Clinical Characteristics of Unilateral Macular Neovascularization Patients with Pachydrusen in the Fellow Eye

**DOI:** 10.3390/jcm13133757

**Published:** 2024-06-27

**Authors:** Hiroyuki Kamao, Erika Mitsui, Yuto Date, Katsutoshi Goto, Kenichi Mizukawa, Atsushi Miki

**Affiliations:** 1 Department of Ophthalmology, Kawasaki Medical School, 577 Matsushima, Kurashiki 701-0114, Okayama, Japan; eri.todo@docomo.ne.jp (E.M.); theethee1103@gmail.com (Y.D.); k_goto@med.kawasaki-m.ac.jp (K.G.); amiki@med.kawasaki-m.ac.jp (A.M.); 2 Shirai Eye Hospital, 1339 Takasecho Kamitakase, Mitoyo 767-0001, Kagawa, Japan; sorararaharururu@gmail.com

**Keywords:** pachydrusen, age-related macular degeneration, anti-vascular endothelial growth factor therapy

## Abstract

**Background/Objectives**: To approach the clinical properties of pachydrusen that differ from conventional drusen, we investigated the incidence of macular neovascularization (MNV) in fellow eyes and the treatment outcomes of intravitreal aflibercept (IVA) in MNV eyes of unilateral MNV patients with pachydrusen in the fellow eye. **Methods**: We retrospectively studied 261 consecutive patients with treatment-naïve unilateral MNV. Patients were classified into four groups according to the type of drusen in the fellow eye: the pachydrusen group (*n* = 49), the soft drusen group (*n* = 63), the subretinal drusenoid deposit (SDD) group (*n* = 24), and the no drusen group (*n* = 125). The development of the MNV in the fellow eye was evaluated for five years, and the retreatment proportion after three monthly aflibercept injections was evaluated for one year. **Results**: The choroidal thickness in the fellow eyes and MNV eyes was the greatest in the pachydrusen group (all *p* < 0.001). The 5-year incidence of MNV in the pachydrusen group was similar to that in the soft drusen group and no drusen group. The pachydrusen group had a lower retreatment rate than the other groups did (pachydrusen group: 46.4%; soft drusen group: 78.1%; SDDs: 87.5%; no drusen group: 83.3%). **Conclusions**: Unilateral MNV patients with pachydrusen in the fellow eye had a lower retreatment rate (46.4%/1 year); therefore, aflibercept monotherapy using the PRN regimen is one of the preferred treatment methods for MNV patients with pachydrusen in the fellow eye.

## 1. Introduction

Macular neovascularization (MNV) secondary to age-related macular degeneration (AMD) is a major cause of visual disturbance in developed countries. Antivascular endothelial growth factor (VEGF) therapy has improved visual and anatomical outcomes in patients with MNV [1]. However, the requirement for frequent administration of expensive anti-VEGF agents causes poor medication adherence [2], and undertreatment poses a risk of visual deterioration [3]. Hence, it is important to identify predictive factors for the efficacy of anti-VEGF therapy in patients with MNV secondary to AMD patients.

Drusen are extracellular materials deposited between the retinal pigment epithelium (RPE) and Bruch’s membrane, and large soft drusen have well-established clinical importance as precursors to neovascular AMD (nAMD) and geographic atrophy [4,5]. Soft drusen contain various components [6,7,8,9], which act as drivers of chronic inflammation that play an important role in the pathogenesis of nAMD. The term “pachychoroid” is a disease entity that systematically defines macular disorder involving a thick choroid that features dilated choroidal vessels in Haller’s layer with attenuated choriocapillaris and choroidal vessels in Sattler’s layer [10]. Pachychoroid disease includes several clinical entities, such as central serous chorioretinopathy (CSC) [11], pachychoroid pigment epitheliopathy (PPE) [10], pachychoroid neovasculopathy (PNV) [12], polypoidal choroidal vasculopathy (PCV) [13], peripapillary pachychoroid syndrome (PPS) [14], and focal choroidal excavation (FCE) [15]. Choroidal abnormalities are associated with progressive dysfunction of RPE, followed by MNV, including PNV. PNV, as well as nAMD, is common in older patients with MNV in Asian countries [16].

Pachydrusen is a relatively new subtype of drusen associated with pachychoroid disease [17]. Pachydrusen are distinctly characterized by an appearance, distribution, and aggregation pattern different from soft drusen. With respect to the efficacy of anti-VEGF therapy for MNV patients, Fukuda et al. showed that the retreatment-free period was longer in polypoidal choroidal vasculopathy (PCV) patients with pachydrusen in the fellow eye than in that with soft drusen in the fellow eye [18]. However, there has been no report on the efficacy of anti-VEGF therapy for MNV patients, including nAMD patients and PNV patients, with pachydrusen in the fellow eye. In this study, we classified the fellow eyes of unilateral MNV patients into four groups: pachydrusen, soft drusen, subretinal drusenoid deposits (SDDs), and no significant drusen (no drusen), and investigated the efficacy of intravitreal aflibercept (IVA) therapy. Moreover, since the natural history of pachydrusen is not fully understood, we investigated the 5-year incidence of MNV in the fellow eye to reveal the clinical importance of pachydrusen in MNV patients.

## 2. Materials and Methods

### 2.1. Study Design

We retrospectively studied 261 consecutive treatment-naïve unilateral MNV patients at Kawasaki Medical School between May 2013 and December 2021. Data regarding hypertension, diabetes, and cigarette smoking were collected from hospital records or patient recall. We divided the patients into never-smokers and ever-smokers, as described in a previous report [19]. We exclude patients who had received or were receiving other anti-VEGF agents (bevacizumab, pegaptanib, ranibizumab, brolucizumab, and faricimab) or had undergone verteporfin photodynamic therapy, laser photocoagulation, or vitrectomy, as were those with MNV as a result of high myopia, uveitis, or angioid streaks. We also excluded patients with eye diseases that could potentially influence the treatment outcome of the studied eye, such as branch retinal vein occlusion, diabetic retinopathy, or glaucoma.

**Figure 1:**
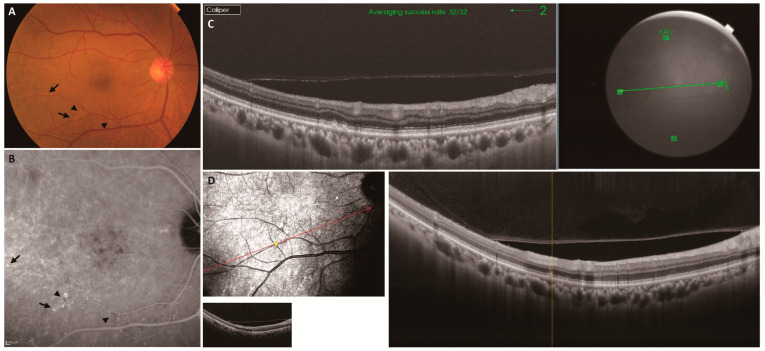
A representative case of the right eye with pachydrusen of a 52-year-old man. (**A**) Color fundus photograph revealed scattered yellowish drusenoid deposits over the temporal lower vascular arcades. No noticeable difference was observed in the appearance of these drusen (arrows and arrowheads). (**B**) The yellowish drusenoid deposits showed a hyperfluorescent area on the late-phase ICGA (arrows). Two yellowish drusenoid deposit presented a hyper-fluorescent area with an interior area of hypofluorescent (arrowheads). (**C**) B-scan with swept-source OCT image corresponding to yellowish drusenoid deposit (arrow) showed sub-RPE accumulation. (**D**) B-scan with spectral domain OCT image corresponding to yellowish drusenoid deposit (arrow) showed sub-RPE accumulation. We observed drusenoid deposits using spectral domain OCT when the fundus images in swept-source OCT were unclear.

We classified the MNV patients into four groups according to the type of drusen in the fellow eye: the pachydrusen group, the soft drusen group, the subretinal drusenoid deposit (SDD) group, and the no significant drusen (no drusen) group. The type of drusen was determined using fundus color photography, near-infrared enface, and spectral domain or swept-source optical coherence tomography (OCT) according to the criteria presented in a previous study [14,20]. Briefly, pachydrusen were defined as yellow-white deposits on color fundus photographs corresponding to homogenous sub-RPE accumulation on OCT images. Pachydrusen had ovoid-shaped deposits with well-defined borders and were isolated or scattered over the posterior pole, typically the temporal vascular arcades. An OCT scan through pachydrusen presented drusenoid deposits detected above the pachyvessels. Pachydrusen typically showed a hyperfluorescent area on the late-phase indocyanine green angiography (ICGA); however, a small part of pachydrusen presented a hyperfluorescent area with an interior area of hypofluorescent (Figure 1). Soft drusen were defined as yellow-white aggregates on color fundus photographs corresponding to homogenous sub-RPE accumulation on OCT images. Soft drusen had round or ovoid shapes with poorly defined borders and were typically tightly packed or even confluent in the macula. Soft drusen showed a hypofluorescent area without a hyperfluorescent area on the late-phase ICGA. We diagnosed the sub-RPE accumulation with hyperfluorescence on late-phase ICGA as pachydrusen and the sub-RPE accumulation with no hyperfluorescence on late-phase ICGA as soft drusen. SDDs were defined as ≥10 discrete whitish deposits on color fundus photographs corresponding to subretinal accumulation on OCT images. Seven eyes with both pachydrusen and soft drusen were classified into the soft drusen group. Twenty-five eyes with both soft drusen and SDDs were classified into the SDD group. No eyes had both pachydrusen and SDDs.

### 2.2. Patients

All patients received three monthly injections (loading dose regimen) of aflibercept and were followed monthly as the pro re nata (PRN) regimen. If retinal exudate (SRF: subretinal fluid and/or IRF: intraretinal fluid) was detected on OCT images after loading dose regimen, all eyes with retinal exudate were treated with the treat-and-extend protocol of IVA therapy. The treatment interval, which was adjusted for 2 to 4 weeks, was reduced when the retinal exudate recurred or persisted and extended when no retinal exudate occurred for six months.

### 2.3. Research and Analysis

All participants underwent a complete ophthalmologic examination, including measurements of best-corrected visual acuity (BCVA), indirect ophthalmoscopy, slit-lamp biomicroscopy with a noncontact lens, color fundus photography and fundus autofluorescence (Canon CX-1; Canon, Tokyo, Japan), spectral domain OCT (RS-3000 Advance OCT; Nidek Corporation, Gamagori, Japan), swept-source OCT (DRI OCT-1 Atlantis; Topcon Corporation, Tokyo, Japan), fluorescein and indocyanine green angiography (HRA-2; Heidelberg Engineering GmbH, Dossenheim, Germany). Visual acuity data were obtained as decimal visual acuity (BCVA) values and converted to logarithm of the minimum angle of resolution (logMAR) units for analysis. The central retinal thickness (CRT) and subfoveal choroidal thickness (SFCT) were measured via swept-source OCT, as described in a previous report [19]. The BCVA, CRT, and SFCT in the MNV eye at baseline and 12 months after initial treatment were measured. We assessed patients for the five-year incidences of progression to MNV in the fellow eye and the one-year outcome of aflibercept monotherapy in the MNV eye. Macular atrophy in the MNV eye at 12 months after initial treatment and geographic atrophy in the fellow eye at 5 years after initial treatment were assessed from two-dimensional cross-sectional OCT images using swept-source OCT. We diagnosed macular atrophy or geographic atrophy in eyes that meet the three diagnostic criteria: choroidal hypertransmission, attenuation of the RPE band, and thinning of the outer retinal layer.

### 2.4. Statistical Analysis

The statistical analyses were performed using JMP Pro 17 software (SAS Institute, Cary, NC, USA). Age, BCVA, CRT, SFCT, and number of afribercept injections received were compared among the four groups by the Kruskal-Wallis test followed by the Steel–Dwass test. Pearson’s chi-square test was used to compare the differences in the female-to-male ratio; incidences of hypertension, diabetes, ever-smokers, and MNV subtypes; incidence of dry macula after the loading dose regimen; incidence of macular atrophy in the MNV eye; and incidence of geographic atrophy in the fellow eye among the four groups. The five-year incidences of progression to MNV in the fellow eye and one-year incidences of retreatment in the MNV eye were constructed by the Kaplan–Meier estimator. A log-rank test was used to compare the differences among the four groups. *p* values < 0.05 were considered to indicate statistical significance in all analyses (* *p* < 0.05; ** *p* < 0.01; † *p* < 0.001).

## 3. Results

### 3.1. Patient Characteristics

In total, 261 eyes of 261 patients with treatment-naïve unilateral MNV were enrolled in our study. The baseline characteristics of the MNV patients were classified into four groups according to the type of drusen in the fellow eye, which were presented in Table 1. The analysis included 49 patients (14 women, 35 men; mean [±SD] age 75.1 ± 7.5 [range, 61–90] years) in the pachydrusen group, 63 patients (22 women, 41 men; mean age 78.0 ± 7.1 [range, 61–92] years) in the soft drusen group, 24 patients (13 women, 11 men; mean age 80.2 ± 7.1 [range, 58–95] years) in the SDD group, and 125 patients (34 women, 91 men; mean age 72.1 ± 9.1 [range, 50–92] years) in the no drusen group. The mean SFCTs of the fellow eyes at the baseline were 236.4 ± 86.5 µm, 188.5 ± 91.8 µm, 126.4 ± 54.2 µm, and 219.4 ± 96.7 µm in the pachydrusen, soft drusen, SDD, and no drusen groups, respectively. The no drusen group was the youngest, and the pachydrusen group had the thickest SFCT among the four groups (age: *p* < 0.001; SFCT: *p* < 0.001). The proportions of patients with typical AMD, PCV, and type 3 MNV were 36.7%, 63.3%, and 0.0%, respectively, in the pachydrusen group; 58.7%, 34.9%, and 6.3%, respectively, in the soft drusen group; 41.7%, 4.2%, and 54.2%, respectively, in the SDD group; and 40.0%, 58.4%, and 1.6%, respectively, in the no drusen group. There was a significant difference in the proportions of patients in the MNV subgroup among the four groups (*p* < 0.001). The pachydrusen group tended to have PCV in the MNV eye. The four study groups were comparable in terms of the female-to-male ratio and proportion of patients with hypertension and diabetes; however, there was a lower proportion of ever-smokers in the SDD group (*p* < 0.03). 

### 3.2. Five-Year Incidence of MNV

**Figure 2:**
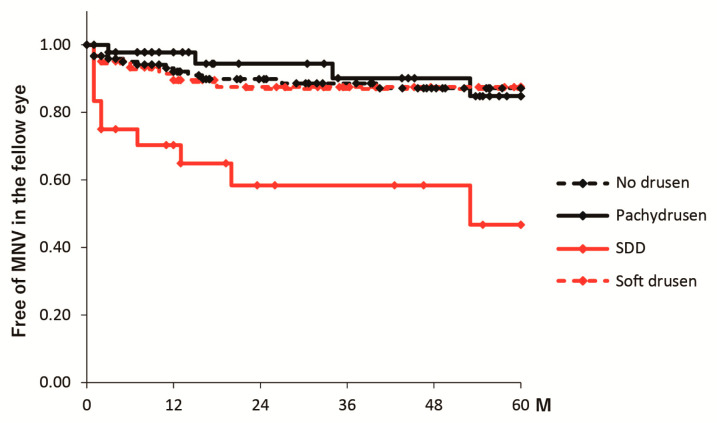
Kaplan-Meier curve for the time to development of the MNV in the fellow eye. The black line represents the pachydrusen group. The black dotted line represents the no drusen group. The red line represents the SDD group. The red dotted line represents the soft drusen group.

Cases that could be followed up for five years were 14 eyes, 34 eyes, 13 eyes, and 60 eyes in the pachydrusen, soft drusen, SDD, and no drusen groups, respectively. The five-year cumulative incidences of progression to MNV in the fellow eye were 15.2% (4 eyes), 12.5% (7 eyes), 53.3% (10 eyes), and 12.9% (13 eyes) in the pachydrusen, soft drusen, SDD, and no drusen groups, respectively (Figure 2). The incidence of progression to MNV was greater in the SDD group than in the other groups (all *p* < 0.001). Among fellow eyes without the development of MNV that were followed up for five years after initial treatment, 0.0% (0 eyes), 10.3% (3 eyes), 25.0% (1 eye), and 3.9% (2 eyes) in the pachydrusen, soft drusen, SDD, and no drusen groups had evidence of geographic atrophy on OCT images, with no significant difference among the groups (*p* = 0.21).

### 3.3. One-Year Outcome of IVA Therapy

Among 306 eyes of 261 MNV patients, including eyes with MNV that developed in the fellow eye during the follow-up, we excluded 170 eyes that were followed up for less than one year after the loading dose regimen with aflibercept. In total, 136 eyes of 125 patients were assessed for the one-year outcome of IVA monotherapy. Twenty-eight eyes in the pachydrusen group, 32 eyes in the soft drusen group, 16 eyes in the SDD group, and 60 eyes in the no drusen group were followed for at least one year after the loading dose regimen with aflibercept (Table 2). The mean BCVA, CRT, and SFCT of the eyes with MNV at the baseline were 0.23 ± 0.28, 319.2 ± 95.9 µm, and 273.7 ± 109.1 µm, respectively, in the pachydrusen group; 0.23 ± 0.32, 328.1 ± 93.3 µm and 224.5 ± 83.4 µm, respectively, in the soft drusen group; 0.44 ± 0.44, 313.0 ± 110.2 µm and 151.27 ± 66.5 µm, respectively, in the SDD group; and 0.27 ± 0.39, 304.5 ± 115.9 µm and 242.0 ± 106.8 µm, respectively, in the no drusen group. There were no significant differences in BCVA or CRT (BCVA: *p* = 0.28; CRT: *p* = 0.48), and the pachydrusen group had the thickest SFCT among the four groups (*p* < 0.001). The four study groups were comparable in terms of the proportion of dry macula cases after the loading dose regimen, the mean change in BCVA, CRT, and SFCT between the baseline and the final visit, and the proportion of macular atrophy cases 12 months after initial treatment (dry macula: *p* = 0.28; BCVA: *p* = 0.08; CRT: *p* = 0.26; SFCT: *p* = 0.96; macular atrophy: *p* = 0.57).

**Figure 3:**
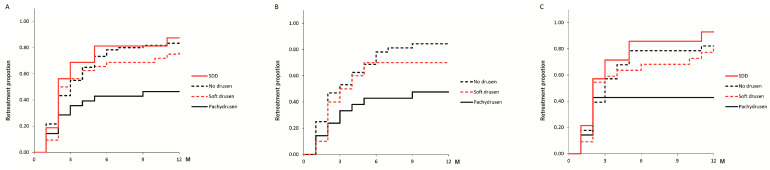
Kaplan-Meier curve for the retreatment proportion after the loading dose regimen. (**A**) All MNV patients. (**B**) MNV patients with polypoidal lesions. (**C**) MNV patients without polypoidal lesions. The black line represents the pachydrusen group. The black dotted line represents the no drusen group. The red line represents the SDD group. The red dotted line represents the soft drusen group. The cases that retreated at one month had retinal exudate one month after the loading dose regimen. In most of these cases, retinal exudate did not disappear during the loading dose regimen. We excluded the SDD group in MNV patients with polypoidal lesions because of the small number.

The retreatment proportions at 12 months after the loading dose regimen were 46.4% (13/28), 78.1% (25/32), 87.5% (14/16), and 83.3% (50/60) in the pachydrusen, soft drusen, SDD, and no drusen groups, respectively. The Kaplan-Meier curve for the retreatment proportion significantly differed among the four groups (*p* < 0.001). The pachydrusen group had a lower retreatment rate than the other groups did (Figure 3A). Furthermore, we classified MNV patients into those with and without polypoidal lesions and investigated the retreatment proportions at 12 months after the loading dose regimen according to the drusen type. Both MNV patients with and without polypoidal lesions demonstrated that the pachydrusen groups had a lower retreatment rate than the other groups [Figure 3B is MNV patients with polypoidal lesions: 47.6% (10/21), 70.0% (7/10), and 84.4% (27/32) in the pachydrusen, soft drusen, and no drusen groups, respectively: *p* = 0.03; Figure 3C is MNV patients without polypoidal lesions: 42.9% (3/7), 81.8% (18/22), 92.9% (13/14), and 82.1% (23/28) in the pachydrusen, soft drusen, SDD, and no drusen groups, respectively: *p* = 0.04].

Cox proportional hazards regression was used to assess the associations between the retreatment proportion and age; the female-to-male ratio; the mean BCVA, CRT, and SFCT at the baseline; and the proportions of hypertension, diabetes, ever-smokers, and the MNV subtype, except for the drusen type, which showed no significant difference (Table 3).

In the pachydrusen group, 13 individuals required retreatment (retreatment group) after the loading regimen, and 18 individuals did not (retreatment-free group) after the loading regimen. The baseline characteristics, including age; female-to-male ratio; mean BCVA, CRT, and SFCT; and proportions of hypertension, diabetes, ever-smokers, and the MNV subtype did not significantly differ between the two groups (Table 4).

## 4. Discussion

Our study revealed that the choroidal thickness was the greatest in the MNV eyes and fellow eyes of the pachydrusen group, and the pachydrusen group tended to have PCV in the MNV eyes and a lower percentage of patients retreated with IVA than did the other groups. Previously, Gass investigated the relationship between nonexudative AMD and subfoveal choroidal thickness [17]; eyes with pachydrusen showed thicker choroid than those with soft drusen or SDD, which is consistent with our results. A previous study by Fukuda et al. classified patients with PCV who received IVA therapy into four groups based on the clinical findings in the fellow eye: the pachydrusen group, the no drusen group, the soft drusen group, and the PCV/scarring group [18]. The authors showed that the retreatment-free period was longer in the pachydrusen group than in the other groups. Although study cohort was different, our findings was consistant with previous results. The effectiveness of anti-VEGF therapy may vary depending on the underlying pathogenesis and age of the patient. In the pathogenesis of MNV, PNV is considered an ischemic disease resulting from dilated choroidal vessels or pachyvessels compressing the immediate overlying choriocapillaris, leading to RPE dysfunction and the development of MNV [21]. Moreover, chronic inflammation caused by soft drusen plays an important role in the pathogenesis of nAMD. Soft drusen contain various inflammatory components, and mutations/polymorphisms in genes coding for complement pathways have been identified as contributors to the pathogenesis of AMD [22,23]. Indeed, a previous study showed that inflammatory cytokine levels in the aqueous humor were lower in PNV patients than in nAMD patients [24]. In the age of MNV patients, PNV patients are younger than nAMD patients are [16,25,26]. Kuroda et al. reported that older age was associated with recurrence after the loading dose regimen in MNV patients treated with anti-VEGF therapy [27]. This study presented that no correlation was found between age and retreatment proportion; therefore, the difference in the mechanism underlying the development of MNV could play a role in the lower retreatment proportion in the pachydrusen group. These findings indicated that anti-VEGF therapy might be more effective in eyes with pachychoroid-driven MNV.

This study revealed that the 5-year incidence of MNV in the fellow eye in the pachydrusen group was similar to that in the soft drusen group. With respect to the incidence of MNV in the fellow eye of unilateral MNV patients, Lee et al., Fukuda et al., and Notomi et al. reported that the pachydrusen group had a lower risk than the soft drusen group [28,29,30], and Teo et al. and Kim et al. reported that the pachydrusen and soft drusen groups were comparable [31,32]. Lee et al. classified patients into five groups using the different classification method as in our study: soft drusen, SDDs, soft drusen + SDDs, pachydrusen, and no drusen; the 5-year incidence of MNV was 7.1% in the pachydrusen group and 46.2% in the soft drusen group. Fukuda et al. classified patients into four groups using the same classification method as in our study, and the 5-year incidence of MNV was 0.0% in the pachydrusen group and 11.1% in the soft drusen group. Fukuda et al. included punctate hyperfluorescent spots on late-phase indocyanine green angiography (ICGA) in their diagnostic criteria for pachydrusen. In a report on punctate hyperfluorescent spots observed via ICGA, Tsujikawa et al. demonstrated that these spots were observed in 93% (38/41) of central serous chorioretinopathy patients on late-phase ICGA [33]. On the other hand, Kang et al. [34] showed that the rate of spatial colocalization of pachydrusen and these spots on late-phase ICGA was 18.2% in eyes with PCV and 0% in eyes with typical AMD; hence, soft drusen are characterized by sub-RPE accumulation without hyperfluorescence in late-phase ICGA. In this study, a large part of pachydrusen showed a hyperfluorescence area on late-phase ICGA; however, a small part of pachydrusen presented a hyperfluorescent area with an interior area of hypofluorescent, as shown in Figure 1. Although the component of pachydrusen has yet to be clarified, the soft drusen comprises various components such as cholesterol, complement, and amyloid-β [35]. The imaging findings could change depending on the size and duration of the drusen; therefore, we diagnosed the sub-RPE accumulation with hyperfluorescence on late-phase ICGA as pachydrusen and the sub-RPE accumulation with no hyperfluorescence on late-phase ICGA as soft drusen. The differences in the classification and diagnostic methods used for drusen may have caused discrepancies between our results and those of other studies. In addition, the AREDS report showed that the 5-year risk of developing nAMD was 30.8%/5 years [4] and 12–50%/5 years [5] in unilateral nAMD patients. Because patients with unilateral MNV were at high risk of developing MNV in their fellow eye, this high risk may have caused no difference in the 5-year development of MNV in the fellow eye between the pachydrusen and soft drusen groups in this study. We found that the 5-year incidence of MNV in the pachydrusen group was similar to that in the soft drusen group; therefore, regular checking of the posterior pole in the fellow eye during anti-VEGF therapy for early diagnosis would benefit unilateral MNV patients.

Anti-VEGF therapy has improved visual outcomes in patients with MNV. Aflibercept [36], widely used as an anti-VEGF agent for patients with MNV, is usually administered at 8–16-week intervals [37], although approximately 30% of MNV patients treated with aflibercept require 4- or 6-week treatment intervals [38,39]. The most significant burden to patients is the need for frequent administration of expensive anti-VEGF agents; therefore, it is important to identify predictive factors for the efficacy of anti-VEGF therapy in patients with MNV. Since anti-VEGF therapy is more effective in PNV patients than in nAMD patients [25,26], it is important to accurately diagnose PNV and nAMD. The choroidal thickness has no definitive cutoff value, because it is correlated with patient age and axial length [40,41]. Hence, no established diagnostic criteria delineate between PNV and nAMD. Our study revealed that the choroidal thickness was the greatest in the MNV eyes and fellow eyes of the pachydrusen group, and the pachydrusen group tended to have PCV in the MNV eyes and a lower percentage of patients retreated with IVA than did the other groups. Pachydrusen and PNV belong to the pachychoroid spectrum; therefore, unilateral MNV patients with pachydrusen in the fellow eye could be diagnosed with PNV.

This study’s limitations include its retrospective nature and relatively small sample size, and we did not evaluate pigment abnormalities as precursor lesions of late AMD. Another limitation is that the proportion of eyes with type 3 MNV evaluated for the efficacy of anti-VEGF therapy was low. In our previous study, 88.2%/year (15/17 eyes) of eyes with type 3 MNV were retreated after the loading dose regimen; thus, we have treated eyes with type 3 MNV using the TAE regimen from the treatment start. Type 3 MNV is associated with SDDs [42], not pachydrusen, and the retreatment proportion in the SDD group in this study was 87.5%/year, which is consistent with our previous results. Previous reports on the retreatment efficacy of IVA therapy using the PRN regimen in Asian patients with unilateral MNV ranged from 63.4% to 73% [43,44,45], and these results are consistent with our results (75.0%). The small proportion of patients with type 3 MNV had a limited impact on the results of this study, namely, the lower proportion of patients who were retreated after the loading dose regimen in the pachydrusen group. Further research is needed to elucidate the mechanism of MNV development because drusen is not the only risk factor for the development of MNV.

## 5. Conclusions

Unilateral MNV patients with pachydrusen in the fellow eye had a lower retreatment rate (46.4%/1 year); therefore, aflibercept monotherapy using the PRN regimen is one of the preferred treatment methods for MNV patients with pachydrusen in the fellow eye.

## Figures and Tables

**Table 1:** Clinical characteristics of unilateral MNV patients.

		Pachydrusen (*n* = 49)	Soft Drusen (*n* = 63)	SDD (*n* = 24)	No Drusen (*n* = 125)	*p*
Age (years), mean (SD)		75.1 (7.5)	78.0 (7.1)	80.2 (7.1)	72.1 (9.1)	<0.001
Sex (female), no. (%)		14 (28.6)	22 (34.9)	13 (54.2)	34 (27.2)	0.06
Hypertension, no. (%)		23 (46.9)	27 (42.9)	14 (58.3)	57 (45.6)	0.63
Diabetes, no. (%)		11 (22.4)	8 (12.7)	4 (16.7)	33 (26.4)	0.20
Smoking habits (ever-smokers), no. (%)		34 (69.4)	39 (61.9)	9 (37.5)	85 (68.0)	0.03
SFCT of the fellow eye (µm), mean (SD)		236.4 (86.5)	188.5 (91.8)	126.4 (54.2)	219.4 (96.7)	<0.001
AMD subtype, no. (%)						<0.001
	Typical AMD	18 (36.7)	37 (58.7)	10 (41.7)	50 (40.0)	
	PCV	31 (63.3)	22 (34.9)	1 (4.2)	73 (58.4)	
	Type 3 MNV	0 (0.0)	4 (6.3)	13 (54.2)	2 (1.6)	

SD: standard deviation, SDD: subretinal drusenoid deposit, SFCT: subfoveal choroidal thickness, AMD: age-related macular degeneration, PCV: polypoidal choroidal vasculopathy, MNV: macular neovascularization.

**Table 2:** One-year outcome of unilateral MNV patients receiving IVA therapy.

		Pachydrusen (*n* = 28)	Soft Drusen (*n* = 32)	SDD (*n* = 16)	No Drusen (*n* = 60)	*p*
Baseline, mean (SD)						
	BCVA (logMAR)	0.23 (0.28)	0.23 (0.32)	0.44 (0.44)	0.27 (0.39)	0.28
	CRT (µm)	319.2 (95.9)	328.1 (93.3)	313.0 (110.2)	304.5 (115.9)	0.48
	SFCT (µm)	273.7 (109.1)	224.5 (83.4)	151.6 (66.5)	242.0 (106.8)	0.001
Final visit, mean (SD)						
	BCVA (logMAR)	0.03 (0.13)	0.10 (0.28)	0.53 (0.73)	0.13 (0.30)	<0.001
	CRT (µm)	211.7 (37.3)	233.8 (45.9)	259.8 (65.4)	221.3 (61.8)	0.02
	SFCT (µm)	241.9 (114.6)	206.4 (109.3)	126.8 (58.2)	206.4 (109.3)	0.003
Change, mean (SD)						
	BCVA (logMAR)	0.20 (0.22)	0.12 (0.26)	−0.09 (0.66)	0.13 (0.34)	0.08
	CRT (µm)	107.7 (98.6)	88.9 (91.5)	53.2 (104.7)	86.9 (125.0)	0.26
	SFCT (µm)	32.0 (33.1)	27.7 (44.5)	24.8 (27.8)	39.1 (57.3)	0.96
Dry macula after loading dose, no. (%)		24 (85.7)	30 (93.8)	13 (81.3)	47 (78.3)	0.28
Additional number of injections, mean (SD)		1.7 (2.2)	3.6 (3.0)	3.1 (3.2)	3.0 (2.4)	0.09
Macular atrophy, no. (%)		7 (25.0)	5 (15.6)	5 (31.3)	11 (18.3)	0.57

SD: standard deviation, SDD: subretinal drusenoid deposit, BCVA: best-corrected visual acuity, CRT: central retinal thickness, SFCT: subfoveal choroidal thickness.

**Table 3:** Cox proportional hazards regression for assessing the association of the retreatment proportion.

		HR (95% CI)	*p*
Age (years)		1.46 (0.57–3.70)	0.43
Sex (female)		1.00 (0.66–1.54)	0.99
Hypertension (yes)		1.04 (0.70–1.54)	0.85
Diabetes (yes)		1.19 (0.78–1.83)	0.42
Smoking habits (ever-smokers)		0.83 (0.55–1.27)	0.40
Drusen type		1.46 (0.57–3.70)	0.03
	Pachydrusen vs. Soft drusen	0.48 (0.25–0.95)	0.03
	Pachydrusen vs. SDD	0.37 (0.17–0.79)	0.01
	Pachydrusen vs. No drusen	0.42 (0.23–0.77)	0.005
	Soft drusen vs. SDD	0.76 (0.40–1.47)	0.42
	Soft drusen vs. No drusen	1.13 (0.62–2.04)	0.55
	SDD vs. No drusen	1.13 (0.62–2.04)	0.69
AMD subtype			0.49
	Typical AMD vs. PCV	1.27 (0.86–1.88)	0.23
	Typical AMD vs. Type 3 MNV	1.08 (0.15–7.80)	0.94
	PCV vs. Type 3 MNV	0.88 (0.11–6.17)	0.87
BCVA, Base line (logMAR)		2.26 (0.51–7.74)	0.24
CRT, Base line (µm)		1.09 (0.20–4.64)	0.92
SFCT, Base line (µm)		0.46 (0.17–1.23)	0.13

HR: hazard ratio, CI: confidence interval, SDD: subretinal drusenoid deposit, AMD: age-related macular degeneration, PCV: polypoidal choroidal vasculopathy, MNV: macular neovascularization, BCVA: best-corrected visual acuity, CRT: central retinal thickness, SFCT: subfoveal choroidal thickness.

**Table 4:** Clinical characteristics of retreatment and retreatment-free group after the loading dose regimen in the pachydrusen group.

		Retreatment (*n* = 13)	Retreatment-Free (*n* = 18)	*p*
Age (years), mean (SD)		75.3 (7.2)	71.9 (6.4)	0.31
Sex (female), no. (%)		4 (30.8)	4 (26.7)	0.81
Hypertension, no. (%)		9 (69.2)	6 (40.0)	0.12
Diabetes, no. (%)		4 (30.8)	2 (13.3)	0.26
Smoking habits (ever-smokers), no. (%)		9 (69.2)	13 (86.7)	0.26
AMD subtype, no. (%)				0.83
	Typical AMD	3 (23.1)	4 (26.7)	
	PCV	10 (76.9)	11 (73.3)	
BCVA, Base line (logMAR)		0.22 (0.27)	0.24 0.29)	0.91
CRT, Base line (µm)		325.5 (114.4)	313.9 (80.2)	0.98
SFCT, Base line (µm)		270.8 (88.4)	276.2 (127.5)	0.93

SD: standard deviation, AMD: age-related macular degeneration, PCV: polypoidal choroidal vasculopathy, BCVA: best-corrected visual acuity, CRT: central retinal thickness, SFCT: subfoveal choroidal thickness.

## Data Availability

The data used to support the findings of this study are restricted by the Kawasaki Medical School Ethics Committee to protect patient privacy. Data are available from Hiroyuki Kamao [hironeri@med.kawasaki-m.ac.jp] for researchers who meet the criteria for access to confidential data.
